# Specific Protein 1 and p53 Interplay Modulates the Expression of the KCTD-Containing Cullin3 Adaptor Suppressor of Hedgehog 2

**DOI:** 10.3389/fcell.2021.638508

**Published:** 2021-04-08

**Authors:** Annapaola Angrisani, Annamaria Di Fiore, Claudia Augusta Di Trani, Simone Fonte, Marialaura Petroni, Ludovica Lospinoso Severini, Fabio Bordin, Laura Belloni, Elisabetta Ferretti, Gianluca Canettieri, Marta Moretti, Enrico De Smaele

**Affiliations:** ^1^Department of Molecular Medicine, Sapienza University, Rome, Italy; ^2^Department of Experimental Medicine, Sapienza University, Rome, Italy; ^3^Department of Internal, Anesthesiological and Cardiovascular Clinical Sciences, Sapienza University of Rome, Rome, Italy; ^4^Istituto Pasteur, Fondazione Cenci-Bolognetti, Sapienza University, Rome, Italy

**Keywords:** KCASH2, KCTD21, Hedgehog, Sp1, p53, medulloblastoma cancer, DNA methylation

## Abstract

The Hedgehog (Hh) signaling pathway plays a crucial role in normal embryonic development and adult tissue homeostasis. On the other end, dysregulated Hh signaling triggers a prolonged mitogenic response that may prompt abnormal cell proliferation, favoring tumorigenesis. Indeed, about 30% of medulloblastomas (MBs), the most common malignant childhood cerebellar tumors, exhibit improper activation of the Hh signaling. The oncosuppressor KCASH2 has been described as a suppressor of the Hh signaling pathway, and low KCASH2 expression was observed in Hh-dependent MB tumor. Therefore, the study of the modulation of KCASH2 expression may provide fundamental information for the development of new therapeutic approaches, aimed to restore physiological KCASH2 levels and Hh inhibition. To this end, we have analyzed the TATA-less KCASH2 proximal promoter and identified key transcriptional regulators of this gene: Sp1, a TF frequently overexpressed in tumors, and the tumor suppressor p53. Here, we show that in WT cells, Sp1 binds KCASH2 promoter on several putative binding sites, leading to increase in KCASH2 expression. On the other hand, p53 is involved in negative regulation of KCASH2. In this context, the balance between p53 and Sp1 expression, and the interplay between these two proteins determine whether Sp1 acts as an activator or a repressor of KCASH2 transcription. Indeed, in p53^–/–^ MEF and p53 mutated tumor cells, we hypothesize that Sp1 drives promoter methylation through increased expression of the DNA methyltransferase 1 (DNMT1) and reduces KCASH2 transcription, which can be reversed by Sp1 inhibition or use of demethylating agents. We suggest therefore that downregulation of KCASH2 expression in tumors could be mediated by gain of Sp1 activity and epigenetic silencing events in cells where p53 functionality is lost. This work may open new venues for novel therapeutic multidrug approaches in the treatment of Hh-dependent tumors carrying p53 deficiency.

## Introduction

The Hedgehog (Hh) signaling pathway plays a crucial role in normal embryonic development and adult tissue homeostasis (Ingham and McMahon, 2001; Quaglio et al., 2020). On the other end, dysregulated Hh signaling triggers a prolonged mitogenic response that may prompt abnormal cell proliferation, favoring tumorigenesis (Pasca di Magliano and Hebrok, 2003; Teglund and Toftgård, 2010).

Indeed, about 30% of medulloblastomas (MBs), the most common malignant childhood brain tumor, and most of the basal cell carcinomas derive from improper activation of the Hh signaling (Raleigh and Reiter, 2019). Furthermore, a growing body of evidences suggests the involvement of Hh in a wider variety of cancers including prostate, gastric, breast, colon, and thyroid cancers (Teglund and Toftgård, 2010; Bushman, 2016; Wu et al., 2017; Xu et al., 2017; Riobo-Del Galdo et al., 2019; Xu et al., 2019), and in cancer cell stemness and multidrug resistance (Sari et al., 2018). Different therapeutic approaches to modulate the Hh pathway have been proposed (Rubin and de Sauvage, 2006; Lospinoso Severini et al., 2020), but the insurgence of mutations that confer tumor resistance is still a critical point, highlighting the need for a multitarget approach, acting on the Hh pathway at different levels (Rubin and de Sauvage, 2006; Lospinoso Severini et al., 2020; Quaglio et al., 2020).

The oncosuppressor KCASH2 (also known as KCTD21) is a suppressor of the Hh signaling pathway and acts through recruitment, ubiquitination, and degradation of the HDAC1 deacetylase, leading to the inhibitory acetylation of the Gli1 transcription factor (De Smaele et al., 2011). Of note, reduced KCASH2 expression (and its related protein KCASH1) has been described in Hh-dependent tumors (De Smaele et al., 2004, 2011; Di Marcotullio et al., 2004), while KCASH2 expression in tumor cells leads to inhibitions of Hh signaling and reduction of tumor cell proliferation (De Smaele et al., 2011; Spiombi et al., 2019).

Based on the above observations, increasing KCASH activity may be a promising tool for therapeutic Hh inhibition. Unfortunately, given the mechanism of action of KCASH2, which acts as an “adaptor molecule,” it appears a challenge to identify small molecules able to improve KCASH2 function. To this end, modulation of KCASH2 protein levels in tumor cells may be a more feasible approach. Therefore, the study of the regulation of KCASH2 expression may provide fundamental information for the development of new therapeutic approaches, aimed to restore physiological KCASH2 levels. Recently, we have identified a KCTD family protein, KCTD15, which has been demonstrated to play a role in the stabilization of KCASH2 protein, therefore increasing its levels and its inhibition on the Hh pathway (Spiombi et al., 2019). Another useful approach to enhance KCASH2 levels is to positively modulate its transcription. To this end, we have aimed our work to the analysis of the KCASH2 proximal promoter for the identification of key transcriptional regulators of this gene, which may be modulated by drugs, either positively or negatively, allowing the activation of this new “physiologic” repressor of the Hh pathway in tumor cells.

## Materials and Methods

### Cell Culture, Transfections, and Treatments

Medulloblastoma cell line DAOY (ATCC HTB-186) was cultured in Minimum Essential Medium (Gibco-Thermo Fisher Scientific, Massachusetts, United States) supplemented with 10% heat-inactivated fetal bovine serum (FBS), 1% sodium pyruvate, 1% non-essential amino acid solution, 1% L-glutamine, and 1% penicillin/streptomycin. DAOY cells were transfected with Lipofectamine Plus, according to the manufacturer’s instructions (Invitrogen-Thermo Fisher Scientific, California, United States).

Wild-type (WT) and p53^–/–^ mouse embryonal fibroblasts (MEF) were generated as described previously (Mauro et al., 2012). Colon cancer cell line HCT116 (ATCC^®^ CCL-247TM), cervical cancer cell line HeLa (ATCC^®^ CCL-2^TM^), HEK293T (ATCC^®^ CRL-3216TM), WT, and p53^–/–^ MEF were cultured in Dulbecco’s modified Eagle’s medium (DMEM, Sigma-Aldrich, St. Louis, United States) plus 10% fetal bovine serum (FBS), 1% L-glutamine, and 1% penicillin/streptomycin, in a 5% CO_2_ atmosphere at 37°C.

HeLa and MEFs were transfected with Lipofectamine 2000, according to the manufacturer’s instructions (Invitrogen-Thermo Fisher Scientific). HEK293T cells were transfected with DreamFect Gold (OZ BIOSCIENCES, San Diego, United States).

Sp1 RNA interference (siRNA) was performed using Silencer^®^ Select Pre-designed siRNA (200 nM), transfected with Lipofectamine 2000 according to the manufacturer’s instructions.

Mycoplasma contamination in cell cultures was routinely screened by using a PCR detection kit (Applied Biological Materials, Richmond, BC, Canada).

Mithramycin A (MMA) was purchased from Sigma (M6891) and reconstituted in methanol to a final concentration of 500 mM for storage. DAOY was treated with MMA (30 nM) for 12 h; HEK293T with 200 nM for 48 h; MEF WT and p53^–/–^ were treated with MMA (500 nM) for 24 h.

DAOY and HCT116 were treated with 5-Aza-2′-deoxycytidine (5-AZA; 5 μM) for 72 h (A3656, Sigma).

HEK293T were incubated with doxorubicin hydrochloride (DOXO; 5 μM; Sigma) for indicated times, and MEF WT and p53^–/–^ cells with DOXO (5 μM) for 5, 10, and 24.

MEF WT were exposed at a 222-nm wavelength of UV-C (10 J/m^2^), using a Strata-linker UV crosslinker 1800 (Stratagene, California, United States). Before UV-C treatment, the culture medium was removed, cells were irradiated, and the medium was added again immediately after irradiation. Cells were then harvested and analyzed after 24 h.

### Plasmids

The following plasmids were used: RSV-Sp1 [kind gift from F. Zazzeroni (Mancarelli et al., 2010)], and pCAG-p53 [carrying mouse wild type p53 (Cecchinelli et al., 2006)]. Human KCASH2 promoter was cloned in pGL4.10 (luc2) (Promega Corporation, Wisconsin, United States) with standard cloning techniques. The mutant KCASH2 promoters were generated using Quick-Change Single Site/Multisite Mutagenesis Kits (Agilent Technologies, California, United States), following manufacturer’s protocols. All constructs were verified by sequencing. The plasmid used for Sp1 siRNA was Silencer^®^ Select Pre-designed siRNA (AM16708, Ambion, Thermo Fisher Scientific, 116546) and as Negative control siRNA #2 (AM4613). Primers are listed in Supplementary Table 1.

### Luciferase Assay

Dual-luciferase assay reactions were prepared using the Firefly Luciferase Assay Kit 2.0 (Biotium, California, United States), following manufacturer’s instructions. Luciferase activity was quantified using GloMax^®^ Discover Microplate Reader (Promega). Results are expressed as Luciferase/Renilla ratios.

### RNA Extraction and RT-qPCR

RNA was extracted using TRizol (Invitrogen-Thermo Fisher Scientific) and RNA Clean and Concentrator^TM^-5 (R1014, Zymo Research, California, United States). cDNA synthesis was performed using the High-Capacity cDNA reverse transcription kit (BIO-65054, Meridian Bioscience, Ohio, United States). Quantitative real-time PCR analysis of KCASH2 mRNA was performed on cDNAs employing TaqMan gene expression assay (Applied Biosystem-Thermo Fisher Scientific) and using the ViiA^TM^ 7 Real-Time PCR System (Applied Biosystem-Thermo Fisher Scientific). All results were normalized to endogenous controls: GAPDH (4310884E), TBP (4326322E), ß2M (4326319E), HPRT, and ß-Actin (4326315E, Applied Biosystem-Thermo Fisher Scientific).

### Chromatin Immunoprecipitation Assay

Chromatin immunoprecipitation assays (ChIP) were performed as previously described (Pediconi et al., 2019).

Briefly, HEK293T cells were fixed in 1% formaldehyde, and they were resuspended in 1 ml of PIPES buffer plus PIC and incubated 10 min at 4°C. Lysates were centrifuged at 10,000 *g* for 5 min to pellet the nuclei. Isolated cross-linked nuclei were sheared by sonication in a 1% SDS lysis buffer to generate cellular chromatin fragments of 300–400 bp using a BioRuptor Sonicator (Diagenode Inc). After microcentrifugation, the supernatant was diluted 1:10 in a buffer 0.01% SDS, 1.1% Triton X-100, 1.2 mM EDTA, 16.7 mM Tris-chloride, pH 8.1, 167 mM NaCl buffer containing protease inhibitors, pre-cleared with blocked Protein G Plus (Pierce), and divided into aliquots. The chromatin was then subjected to immunoprecipitation for 14–16 h at 4°C using antibodies specific to anti-Sp1 (ab227383; Abcam), anti-acetyl-H4 (06-866; Millipore, Burlington, Massachusetts, United States), and anti-p53 (#2524A; Cell signaling). Immunoprecipitations with non-specific immunoglobulins (#27478; Abcam) were included in each experiment as a negative control. After the reverse cross-linking, immunoprecipitated chromatin was purified by phenol/chloroform (1:1) extraction and ethanol precipitation and analyzed by real-time PCR amplification using primers for KCASH2 promoter (listed in Supplementary Table 1).

### Oligo Pulldown Assay

Nuclear extracts were prepared with NE-PER Nuclear and Cytoplasmatic Extraction reagents (Thermo Fisher Scientific, Pierce Biotechnology, Rockford, Illinois, United States) according to the manufacturer’s instructions and stored at −80°C.

Double-strand-biotinylated oligonucleotides were prepared using an equal quantity of single-stranded sense and antisense biotinylated oligonucleotides heated in a 100°C water bath for 1 h and allowed to cool down at RT.

The pulldown was performed with Dynabeads MyOne Streptavidin C1 (Invitrogen-Thermo Fisher Scientific) following manufacturer’s instruction. Briefly, 100 μl of resuspended washed Dynabeads magnetic beads was added to a mix formed by 400 μg of Nuclear extract and 4 μg of double-strand-biotinylated oligonucleotide in 100 μl of PBS buffer and placed on a rocking platform for 2 h. Then, the biotinylated oligonucleotide-coated beads were separated from the mix with a magnet for 3 min. Following washes, beads were resuspended in 30 μl of Loading Buffer 2×, boiled for 5 min at 95°C, separated from the supernatant with a magnet for 3 min, and analyzed by Western blot. Biotinylated probes are listed in Supplementary Table 1.

### Western Blot

Cells were lysed with buffer containing Tris-HCl pH 7.6 (50 mM), 1% deoxycholic acid sodium salt, NaCl (150 mM), 1% NP40, EDTA (5 mM), NaF (100 mM), supplemented with phosphatase inhibitor, and Halt Protease Inhibitor cocktail (Thermo Fisher Scientific). Total protein extracts were then evaluated by Western blot assay using the antibodies listed below: mouse anti-tubulin polyclonal (SC-8035; Santa Cruz Biotechnology, Heidelberg, Germany), mouse monoclonal antibody against ß-actin (AC-15, A5441, Sigma), mouse anti-Vinculin monoclonal (SC-73614; Santa Cruz Biotechnology), mouse anti-GAPDH (6C5) (ab8245 Abcam, Cambridge, United Kingdom), rabbit anti-KCTD21/KCASH2 monoclonal (ab192259; Abcam), rabbit polyclonal anti-Sp1 (ab227383; Abcam), rabbit polyclonal anti-Phospho-p53 (Ser15; #9284, Cell Signaling Technology, Massachusetts, United States), and rabbit polyclonal anti-p53 (#9282, Cell Signaling). HRP-conjugated secondary antibody anti-mouse (SC-516102) or anti-rabbit (SC-2357) was purchased from Santa Cruz.

### DNA Methylation Assay

Total genomic DNA was isolated from DAOY and HCT116 cells using the Isolate II Genomic DNA Kit (BIO-52067, Meridian BIOSCIENCE, Memphis, Tennessee United States). Four hundred nanograms of total DNA was treated with bisulfite to convert unmethylated cytosine to uracil using the Protocol A of EpiJET Bisulfite Conversion Kit (K1461, Thermo Fisher Scientific).

Either methylated or unmethylated DNA was used for PCR amplification carried out using Phusion U Hot Start DNA Polymerase (Thermo Fisher Scientific, F-555S/L). PCR primers were designed by the EpiDesigner software^1^ and are listed in Supplementary Table 1.

The amplificated DNAs were then sequenced using the Sanger method [as previously reported (Belardinilli et al., 2015; Nicolussi et al., 2019)]. Sequencing was performed using the BigDye Terminator v3.1 Cycle Sequencing Kit and a 3130XL Genetic Analyzer (Applied Biosystems–Thermo Fisher Scientific). Sequences were analyzed with the 4Peaks software (Nucleobytes, Aalsmeer, The Netherlands). The methylation level was measured as a ratio of methylated CpG to total CpG.

### *In silico* Promoter Analysis

The human and mouse proximal promoter sequence of KCASH2 was identified using Promoter MatInspector (Genomatix software) (Cartharius et al., 2005). The Transcription Start Site (TSS) was recognized using the UCSC Genome Browser^2^. The putative transcription factor binding sites were identified using the Promoter MatInspector, GeneXPlain-Transfac^3^, and JASPAR database (Fornes et al., 2020). CpG islands prediction was performed with EMBOSS CpG-plot and MethPrimer (Li and Dahiya, 2002; Madeira et al., 2019). Promoter sequences of human and mouse KCASH2 were aligned by using the EMBL software Clustal Omega (Madeira et al., 2019).

### Statistical Analysis

For all luciferase and qPCR assays, the *p*-values were determined using Student’s *t*-test, and statistical significance was set at *^∗^p* < 0.05, *^∗∗^p* < 0.01, or *^∗∗∗^p* < 0.001. Results are expressed as mean ± SD. All experiments were replicated biologically at least three times.

## Results

### Description of KCTD-Containing Cullin3 Adaptor Suppressor of Hedgehog 2 Proximal Promoter

Analysis of the proximal promoter is a fundamental step to identify regulatory networks involved in the expression of a specific gene, in particular, physio-pathological contexts (Veerla and Höglund, 2006).

We identified the human proximal promoter of KCASH2, using the Promoter Inspector Genomatix software. The Transcription Start Site (TSS) was recognized using the UCSC Genome Browser. KCASH2 proximal promoter is an 874-bp region that ends 169 bp downstream to the TSS and is a TATA/CAAT-less promoter.

**FIGURE 1:**
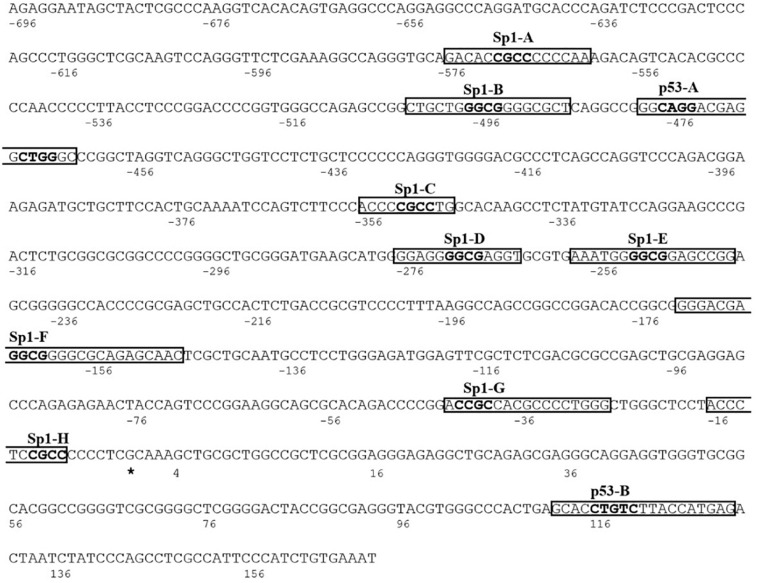
The human KCTD-containing Cullin3 Adaptor of Suppressor of Hedgehog 2 (KCASH2) proximal promoter. The Transcription Start Site (TSS) is identified with the asterisk and is considered as nucleotide number 1. The putative binding sequences for Sp1 and p53 TFs are named from A to H and A to B, respectively. The relative core sequences are highlighted in bold.

To identify *cis*-acting elements in the promoter region responsible for KCASH2 regulation, we performed computational analysis, selecting the most significant transcription factor (TF) binding sites (BSs) (Supplementary Figure 1). Among these, we identified eight Specific Protein 1 (Sp1) BS sequence, named Sp1A to Sp1H (Figure 1). Sp1, a well-known TF ubiquitously expressed in mammalian cells, binds to, and acts through, GC boxes on the promoter of multiple target genes lacking a TATA box, which are involved in several fundamental cellular functions, including a number of tumor suppressors (Beishline and Azizkhan-Clifford, 2015). Since KCASH2 promoter lacks TATA and CAAT sequences, and has several GC boxes, we hypothesized that Sp1 could play an important role in KCASH2 regulation.

Interestingly, we also identified on KCASH2 promoter two putative BS (named p53A and p53B) for the tumor suppressor p53 (Figure 1). p53 regulates several genes, coordinating essential biological processes from cell cycle progression to DNA repair up to senescence and apoptosis, which, if altered, underlie malignant transformation (Lane and Levine, 2010). Indeed, when p53 function is lost, normal cells lose their ability to control growth and death, leading to uncontrolled proliferation and cancer (Vieler and Sanyal, 2018). Since over 50% of human tumors carry loss of function mutations of p53 (Vieler and Sanyal, 2018), it appears useful to understand if and how p53 is able to regulate KCASH2 transcription.

To study *in vitro* the transcriptional modulation of the KCASH2 proximal promoter, we cloned the promoter into a luciferase reporter vector [pGL4.10 (luc2)], upstream of the reporter. After transfection of the KCASH2 reporter gene in HEK293T cells, we observed that the luciferase activity driven by the KCASH2 promoter is 24-fold over the background activity of the empty pGL4.10-basic (Supplementary Figure 2). Thanks to its substantial basal activity, the luciferase reporter looked suitable for analyzing both positive and negative transcriptional modulation of KCASH2 and, therefore, the effects of ectopic expression of different TFs on the KCASH2 promoter.

### SP1 Activates Basal Transcriptional Activity of KCASH2 Promoter

**FIGURE 2:**
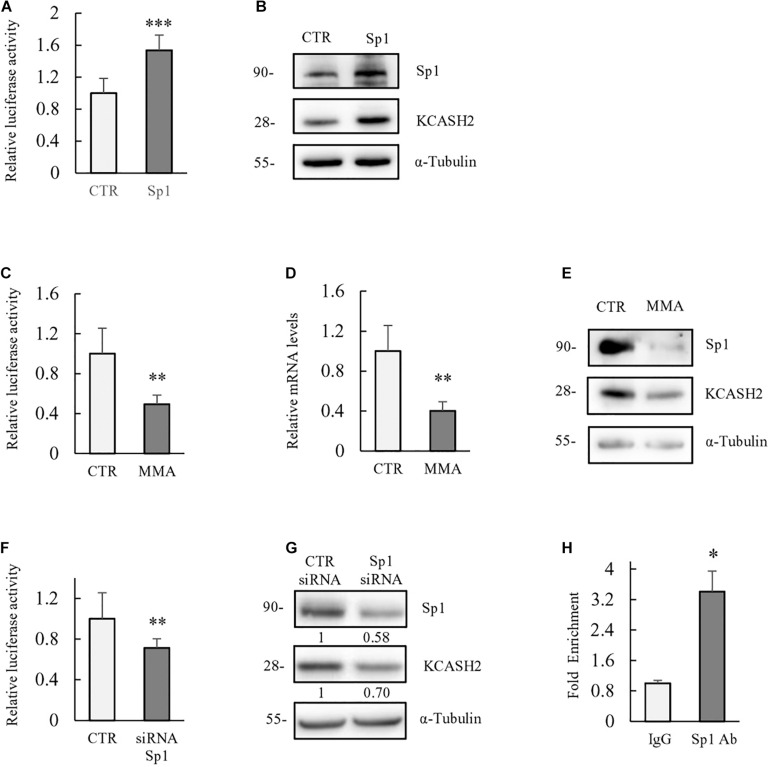
Basal transcriptional activity of human KCASH2 promoter is driven by Sp1. **(A,B)** Sp1 ectopic expression increases KCASH2 promoter activity and KCASH2 protein levels. **(A)** HEK293T were co-transfected with the KCASH2 promoter luciferase reporter, together with control (CTR) vector or Sp1 expressing plasmid. Luciferase activity was normalized to the control. ^∗∗∗^*p* < 0.001. **(B)** HEK293T were transfected with empty vector or Sp1 expressing vector; 24 h later, cells were lysed and analyzed by Western blot, using Sp1 and KCASH2 antibodies. Tubulin was used as loading control. **(C–G)** Inhibition of Sp1 leads to a decrease in KCASH2 reporter activity, KCASH2 mRNA, and protein levels. **(C)** HEK293T cells were co-transfected with KCASH2-Luc reporter and treated with MMA (200 nM) for 24 h. Luciferase activity was normalized to mock treated cells (CTR). ^∗∗^*p* < 0.01. **(D,E)** HEK293T cells were cultured with MMA (200 nM) for 48 h. Then, cells were lysed and KCASH2 mRNA **(D)** and protein levels **(E)** were evaluated by RT-qPCR and Western Blot, respectively. KCASH2 mRNA levels were normalized to GAPDH, TBP, ß2M, and represented as fold-induction of the CTR. ^∗∗^*p* < 0.01. Western Blot was performed as above. Transfection efficiency in luciferase experiments was normalized by co-transfection of a pRL–TK–Renilla reporter. All experiments represented are the mean of three independent experiments ± standard deviation (SD). **(F)** Relative luciferase activity was measured in HEK293T cells transfected with scrambled siRNA (siCTR) or with Sp1 siRNA followed by KCASH2-Luc reporter and pRL–TK Renilla. Luciferase activity was normalized to the control. ^∗∗^*p* < 0.01. **(G)** HEK293T were transfected with siCTR or with Sp1 siRNA, 24 h later, cells were lysed and analyzed by Western Blot, using Sp1 and KCASH2 antibodies. Tubulin was used as loading control. Band intensities were analyzed using image J software and numbers below the boxes represent the relative quantification of protein levels. **(H)** Sp1 binds *in vivo* to the KCASH2 promoter sequence. Cross-linked chromatin was extracted from HEK293T cells and immunoprecipitated with a relevant control IgG or specific anti-Sp1 antibody. Immunoprecipitated chromatin samples were analyzed by qPCR using KCASH2 promoter selective primers. Relative enrichment was calculated by Delta CT analysis and expressed as fold induction of immunoprecipitated IgG negative control vs. specific anti-Sp1 antibody. ^∗^*p* < 0.05.

To evaluate Sp1 contribution to KCASH2 transcription, we performed luciferase assay in HEK293T cells co-transfected with the KCASH2 reporter, in the presence of control vector or plasmid expressing Sp1. As shown, Sp1 overexpression significantly increases the KCASH2 reporter luciferase activity (Figure 2A) and KCASH2 protein levels (Figure 2B).

Moreover, we treated HEK293T cells with MMA (200 nM), a potent anticancer drug that binds to the minor grooves of the GC-rich motifs of the DNA, displacing Sp1 activity selectively (Quarni et al., 2019). As expected, luciferase assay shows a decrease in KCASH2 promoter activity following MMA treatment (Figure 2C). Similarly, mRNA and protein levels of KCASH2 significantly decrease in HEK293T cells after 48 h of drug treatment (Figures 2D,E), confirming Sp1-positive regulation of KCASH2 transcription. To further confirm that the KCASH2 transcription is indeed sustained by Sp1, we monitored also the effect of the depletion of endogenous Sp1 on KCASH2 activity. As expected, siRNA-mediated depletion of endogenous Sp1 reduces luciferase activity of KCASH2 promoter (Figure 2F) and its protein levels (Figure 2G) at similar levels. Finally, we confirmed the *in vivo* binding of Sp1 on KCASH2 gene promoter, performing a ChIP-qPCR assay in HEK293T cells by using anti Sp1 antibody (Figure 2H). The increase in the amount of immunoprecipitated proximal KCASH2 promoter confirms *in vivo* Sp1 binding, which most likely sustains its basal activity.

Next, we analyzed the contribution of each of the eight putative Sp1 BS (labeled from Sp1A to Sp1H) to KCASH2 regulation. Indeed, this contribution may depend on their relative position and the distance between them and the BS of other TFs, which can determine the availability for activators or co-repressor to interact with Sp1 (Koutsodontis et al., 2001).

**FIGURE 3:**
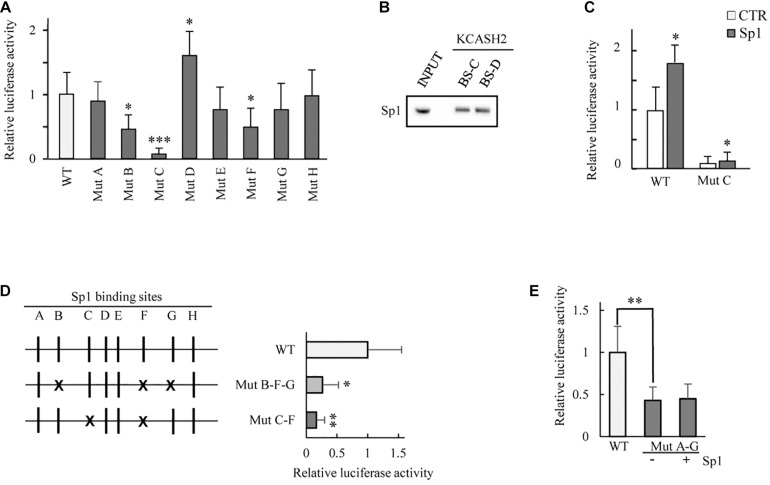
Analysis of the contribution of each Sp1 BS on KCASH2 transcription. **(A)** HEK293T cells were co-transfected with wild type KCASH2 promoter sequence (WT) or with one of eight luciferase reporters (named Mut A to H), each carrying a mutated and inactive Sp1 BS. Lysates were analyzed by luciferase assays. ^∗^*p* < 0.05; ^∗∗∗^*p* < 0.001. **(B)** Oligo pull-down assay. The pulldown was performed with Dynabeads magnetic beads added with 400 μg of HEK293T Nuclear extract and 4 μg of double strand-biotinylated-oligonucleotide. INPUT: 40 μg of Nuclear extract. **(C)** HEK293T were transfected with WT or Mut C luciferase reporter together with an empty vector or a SP1 expressing vector. Lysates were analyzed by luciferase assays. ^∗^*p* < 0.05. **(D)** HEK293T were co-transfected with WT promoter or alternatively with Mut B/F/G or Mut C/F luciferase vectors. Lysates were analyzed by luciferase assays. ^∗^*p* < 0.05; ^∗∗^*p* < 0.01. **(E)** Mutations from A to G Sp1 BS reduce significantly KCASH2 activity and render the promoter unable to respond to Sp1 overexpression. HEK293T cells were co-transfected with WT reporter or Mut A–G luciferase vector, together with CTR or Sp1 expressing plasmids. Lysates were analyzed by luciferase assays. ^∗∗^*p* < 0.01. Transfection efficiency in luciferase experiments was normalized by co-transfection of a pRL-TK-Renilla reporter. Data are representative of three independent experiments performed in triplicate and presented as mean ± SD.

We generated eight luciferase KCASH2 reporter constructs, each carrying a mutation disrupting the core sequence of one Sp1 BS (named MutA to MutH). The KCASH2 luciferase activity is significantly reduced when Sp1B and Sp1F have been mutated (MutB and MutF), and this effect was even more pronounced following the mutation of Sp1C (MutC). Overall, the other site-specific mutations lead to a modest and statistically non-significant decrement in KCASH2 activity. The only exception is represented by the fourth Sp1 BS mutant (MutD), which induces a significant upregulation of KCASH2 luciferase activity (Figure 3A). Since the opposite effects of mutations of sites C and D may be due to differences in the actual transcription factors that bind them, we performed oligonucleotide pulldown assays, observing that both sequences are able to bind Sp1 with similar affinity (Figure 3B).

We also evaluated the residual responsiveness of MutC, for which we obtained the most marked decrease in basal activity, to Sp1 overexpression. Of note, a residual responsiveness of MutC to Sp1 is still present (increasing about 50% above the basal), indicating that although Sp1C BS has a dominant role in the modulation of KCASH2 by Sp1 (Figure 3C), nevertheless, some residual responsivity due to the other BS remains.

Given the most relevant role of Sp1B, Sp1C, and Sp1F in the KCASH2 promoter, we performed multiple mutagenesis to evaluate the contribution of different combinations of these BS. In particular, one mutant carrying Sp1B, Sp1F, and Sp1G mutations lead to a decrease in KCASH2 reporter activity by a value consistent with the summatory of addition of the effects observed in the presence of single Sp1 BS mutations, while a mutant adding Sp1F mutation to Sp1C mutation did not further reduce the basal activity compared with MutC (Figure 3D).

Finally, we evaluated the activity of KCASH2 reporter with mutations in all Sp1 BS, except for the site Sp1H (whose mutation does not affect reporter activity, which remained substantially overlapping with WT reporter) (MutA-G; Figure 3E). The mutation of Sp1D, which may be involved in an increase in KCASH2 activity, balances, in part, the negative effects of the other mutations, resulting in a higher basal luciferase activity of the MutaA-G than of the multiple Sp1B/F/G and Sp1C/F mutants. This seems to indicate that the Sp1D site may be physiologically involved in the suppression of KCASH2 transcription by a different TF (or a cofactor) sitting on the actual sequence or on a nearby sequence and interacting with Sp1 TF. Interestingly, the ectopic expression of Sp1 does not increase the MutA-G reporter activity, indicating that the identified BSs are the ones responsible for KCASH2 responsivity to Sp1 (Figure 3E).

### p53 Negatively Regulates KCASH2 Expression

Two putative p53 BSs were recognized on human KCASH2 promoter sequence. The first map from −480 to −460 bp and the second from 112 to130 bp (downstream of the TSS). The second p53BS is not conserved in the mouse KCASH2 promoter and was not investigated further (Supplementary Figure 3).

**FIGURE 4:**
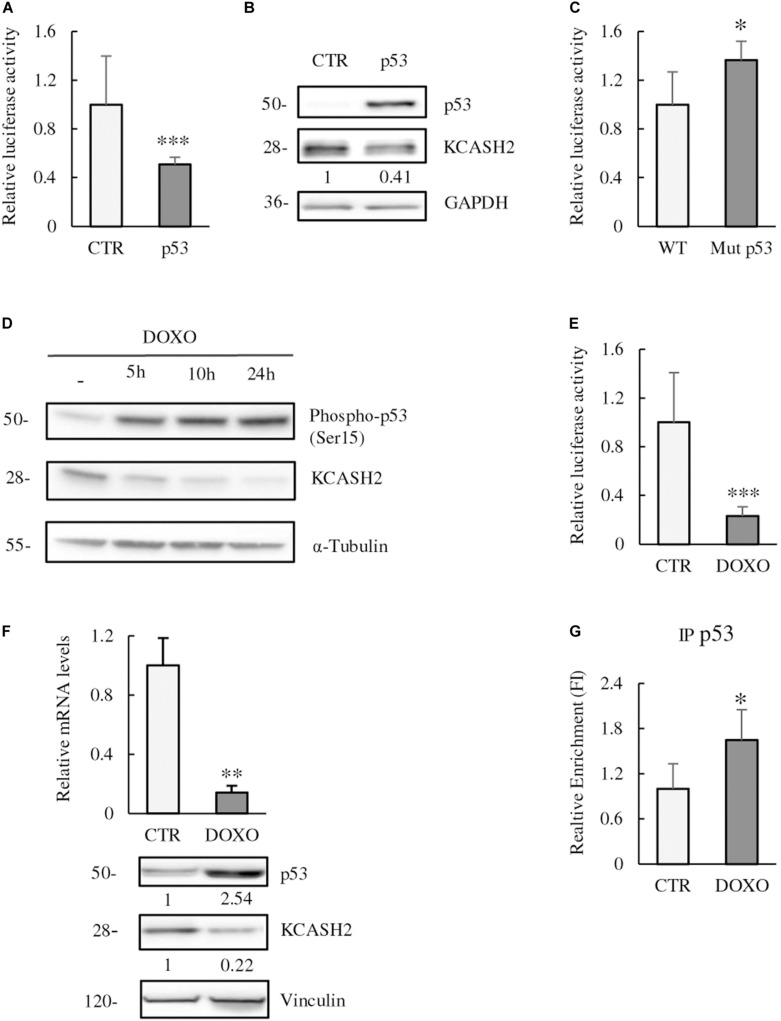
p53 is a negative regulator of KCASH2 transcription. **(A,B)** p53 ectopic expression leads to a decrease in KCASH2 activity. **(A)** HEK293T cells were co-transfected with KCASH2-Luc reporter, together with an empty vector or a p53 expressing vector. Lysates were analyzed by luciferase assays. ^∗^*p* < 0.05. **(B)** HEK293T cells were transfected with p53 expressing or empty vector. Protein lysates were analyzed by Western blot, using p53 and KCASH2 antibodies. GAPDH was used as loading control. **(C)** KCASH2 promoter mutated on the p53 BS loses responsiveness to p53. HEK293T were co-transfected with WT or Mut p53 luciferase vector. Lysates were analyzed through luciferase assays. ^∗^*p* < 0.05. **(D–F)** Increase of p53 activity leads to a reduced KCASH2 expression. **(D)** HEK293T cells were treated with DOXO (5 μM) and collected at different time points (5, 10, and 24 h). Western blot analysis of p53 activation was performed with an antibody against phosphorylation of its Ser15, KCASH2 levels with specific antibody. Anti-Tubulin was used as loading control. **(E)** Luciferase assay of HEK293T cells co-transfected with KCASH2-Luc reporter and treated with DOXO (5 μM) for 24 h. ^∗∗∗^*p* < 0.001. **(F)** Modulation of KCASH2 mRNA and protein levels following treatment with DOXO (5 μM) for 10 h. mRNA (upper panel) was assayed through RT-qPCR, normalized to GAPDH and Actin, and represented as fold induction on the CTR. ^∗∗^*p* < 0.01. The corresponding levels of p53 and KCASH2 proteins (lower panel) were analyzed by Western blot. Anti-Vinculin was used as loading control. Transfection efficiency in luciferase experiments was normalized by co-transfection of a pRL–TK–Renilla reporter. Data are representative of three experiments performed in triplicate and presented as mean ± SD. **(G)** p53 binds *in vivo* KCASH2 promoter. Chromatin IP assay was performed with control IgG or anti-p53 antibody. Cross-linked chromatin was extracted from HEK293T cells treated for 5 h with Doxorubicin 5 μM and immunoprecipitated. Immunoprecipitated chromatin samples were analyzed by qPCR using KCASH2 promoter selective primers. Relative enrichment was calculated by Delta CT analysis and is expressed as fold induction of treated vs. not treated. ^∗^*p* < 0.05.

We examined the p53 contribution to KCASH2 transcriptional regulation. Following p53 overexpression, KCASH2 reporter activity was significantly downregulated in HEK293T cells (Figure 4A). Similarly, KCASH2 protein levels decrease in p53-overexpressing cells (Figure 4B).

To verify whether p53 was acting through the predicted p53 BS, we mutated the core sequence of the recognition site for p53. As expected, Mutp53 reporter presented a significantly higher activity compared with WT promoter in luciferase assays, confirming that p53 BS is fundamental for p53 to perform its function (Figure 4C).

We evaluated also the effect of p53 modulation on KCASH2 expression. For this purpose, we treated HEK293T cells at different time points (5, 10, and 24 h) with DOXO (5 μM), a chemotherapic drug that causes DNA double-strand breaks and p53 activation (Lin et al., 2010). DOXO exposition leads to p53 activation [detected by p53 phosphorylation at Ser15 (Loughery et al., 2014)], and a concomitant decrease in KCASH2 protein levels as early as 5 h after treatment (Figure 4D). Similarly, DOXO treatment leads to a significant reduction in KCASH2 reporter activity (Figure 4E) and KCASH2 mRNA and protein expression (Figure 4F, upper and lower panels, respectively). Next, to determine whether active p53 could bind to the KCASH2 promoter sequence, ChIP assays were conducted in HEK293T cells where p53 was activated by DOXO treatment. Results revealed that p53 can directly bind to the KCASH2 promoter (Figure 4G). Interestingly, following DOXO treatment and p53 activation, the transcriptional activity on KCASH2 promoter is reduced. In fact, ChIP experiment with Anti-Acetyl-H4 (acH4) antibody, an epigenetic marker of transcriptional activation, demonstrated a general decrement of KCASH2 chromatin accessibility and a transcriptional inhibition of KCASH2 gene (Supplementary Figure 4).

Since p53 and Sp1 share similar consensus sequences at GC-boxes along the human genome, it is known that they might interplay in transcription regulation or compete in binding to promoters and function in opposite directions (Thornborrow and Manfredi, 2001; Lin et al., 2010; Oppenheim and Lahav, 2017). We wondered therefore if the apparent paradox of the opposite effects of the mutations in sites C and D on KCASH2 promoter (Figure 3A) may be due to the fact that site C could be bound by the activator member of Sp1 family, and site D could instead be occupied by p53.

With this aim, we performed an oligo pulldown assay that indicated that p53 is not able to bind efficiently the C binding site (BS-C) or the D binding site (BS-D) (Supplementary Figure 5A). Analysis of the luciferase activity of KCASH2 reporter containing either the mutation in the C binding site or the D binding sites (MutC or MutD), in presence of ectopic p53, did not suggest a significant difference in the effect of p53 (Supplementary Figure 5B). Indeed, p53 expression leads to a similar decrease in transcriptional activity in both WT and mutant constructs. These data may be interpreted as an indication that p53 activity on the KCASH2 promoter does not requires the Sp1 sites C and D.

### p53 and Sp1 Regulation of KCASH2 Transcription Is Conserved in Mouse

Using MatInspector, we extrapolated the mouse KCASH2 proximal promoter sequence, and we compared it with the human promoter sequence, through Clustal Multiple Software (see Supplementary Figure 3). The observed high homology (∼70%), in particular, in the regions corresponding to Sp1 and p53 BS (that are highly conserved) suggests the presence of a largely conserved regulatory mechanism.

**FIGURE 5:**
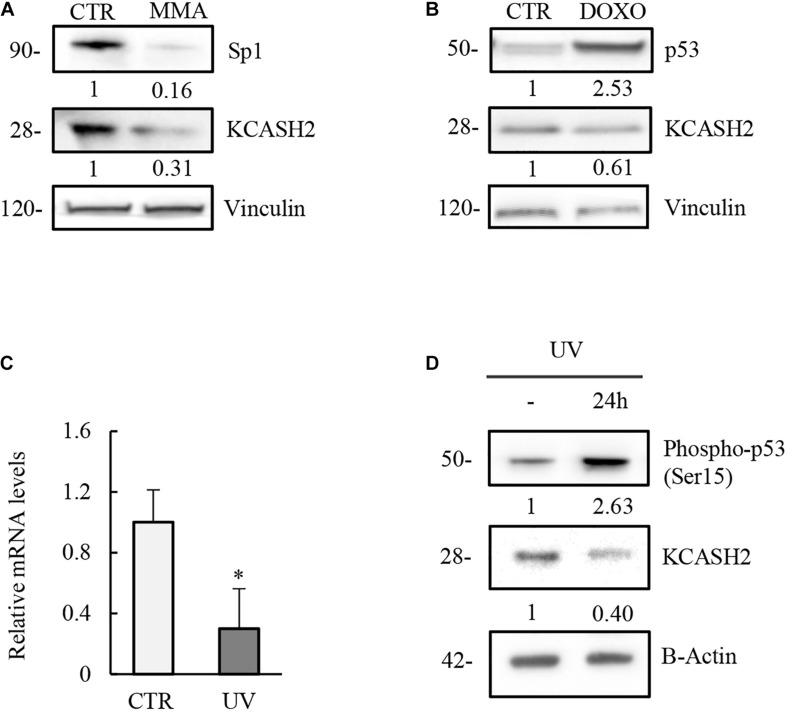
Modulation of KCASH2 expression by p53 and Sp1 is conserved in mouse. **(A)** Inhibition of Sp1 leads to a decrease of KCASH2 protein levels. MEF cells were treated with MMA (500 nM) for 24 h. Protein lysates were analyzed by Western Blot, using Sp1 and KCASH2 antibodies. Anti-vinculin was used as loading control. **(B–D)** p53 downregulates KCASH2 expression. **(B)** MEF cells were cultured with DOXO (5 μM) for 10 h, then cells lysates were analyzed by Western blot. Vinculin was used as loading control. **(C,D)** MEF cells were exposed to UV-C light and collected after 24 h. Then, RT-qPCR analysis was performed **(C)**. Endogenous KCASH2 mRNA levels were normalized on GAPDH and HPRT, and represented as fold-induction on CTR. Data are representative of three experiments and presented as mean ± SD. ^∗^*p* < 0.05. Endogenous KCASH2 protein and p53 activation **(D)** were analyzed by Western blot and revealed with antibodies against KCASH2 and Ser15 phosphorylated p53. Anti-β-actin is shown as loading control.

To verify this hypothesis, we assessed the Sp1 role on mouse KCASH2 expression by treating mouse embryonal fibroblast (MEF) with MMA. As expected, interfering with the Sp1 activity leads to a decrease in KCASH2 protein levels (Figure 5A).

Similarly, activation of p53 is responsible for a decrease in KCASH2 levels in MEF cells, after both DOXO treatment (Figure 5B) and UV irradiation (mRNA and protein levels; Figures 5C,D).

### Sp1 Negatively Regulates KCASH2 Promoter in p53-Deficient Cells

Given that p53 and Sp1 work similarly in human and mouse cell lines, in order to evaluate a potential interplay between the two factors, we decided to use immortalized MEF generated from a p53^–/–^ mouse model as a tool to investigate the effect of Sp1-driven transcription of KCASH2 in the absence of p53.

**FIGURE 6:**
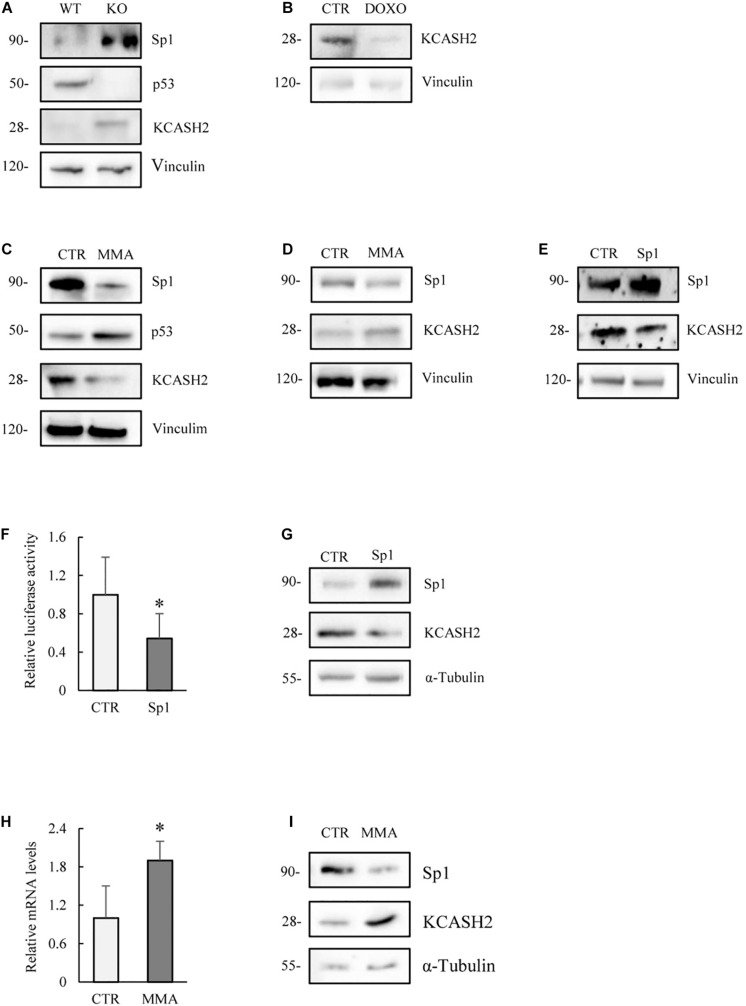
Analysis of the modulation of KCASH2 expression in cells lacking p53 activity. **(A)** MEF p53^–/–^ present increased endogenous Sp1 and KCASH2 protein levels. Protein lysates of MEF WT and p53^–/–^ were analyzed by Western blot. Anti-Vinculin is shown as loading control. **(B)** DOXO treatment reduces KCASH2 protein levels in MEF p53^–/–^. MEF p53^–/–^ cells were treated with DOXO (5 μM) for 10 h, then cells were lysed and analyzed by Western blot. Anti-Vinculin was used as loading control. **(C)** MMA treatment depletes KCASH2 protein levels and increases p53 stability in MEF WT. MEF WT were cultured with MMA (500 nM) for 24 h, then protein lysates were analyzed by Western blot. Anti-Vinculin is shown as loading control. **(D,E)** Sp1 negatively regulates KCASH2 in MEF p53^–/–^. **(D)** MEF p53^–/–^ were treated with MMA (500 nM) for 24 h and protein lysates analyzed by Western blot. Anti-Vinculin was used as loading control. **(E)** MEF p53^–/–^ were transfected with empty vector or Sp1 expressing plasmid, then protein lysates analyzed by Western blot. Vinculin was used as loading control. **(F,G)** Sp1 overexpression reduces KCASH2 activity in p53 mutant DAOY cells. **(F)** DAOY were co-transfected with KCASH2-Luc reporter, together with control vector or Sp1 plasmid expression. Luciferase activity is normalized to the control and represented as mean of three independent experiments ± SD. ^∗^*p* < 0.05. **(G)** DAOY were transfected with empty vector or Sp1 expressing vector. Protein lysates were assayed by Western Blot. Tubulin was used as loading control. **(H,I)** Sp1 inhibition leads to an increase of KCASH2 mRNA and protein levels. **(H)** DAOY cells were cultured with MMA (30 nM) for 12 h. KCASH2 mRNA levels were assayed through RT-qPCR, normalized to GAPDH, HPRT, and Actin, and represented as fold-induction on the CTR. Bars represent the mean of three independent experiments ± SD. ^∗^*p* < 0.05. **(I)** Following MMA treatment, DAOY protein lysates were analyzed by Western Blot. Tubulin was used as loading control.

Comparison of the endogenous protein levels in MEF WT and p53^–/–^ cells indicates that p53 absence matches with an increase in both Sp1 and KCASH2 proteins (Figure 6A). Indeed, our data are coherent with previous observations that p53 may negatively regulate Sp1 (Zhang et al., 2006).

Interestingly, DOXO treatment reduces KCASH2 protein levels also in MEF p53^–/–^ (Figure 6B), indicating a p53-independent route of action. Indeed, other groups have shown that exposure to DOXO leads to reduced protein levels of Sp1 levels in time- and dose-dependent manners (Oppenheim and Lahav, 2017), which could explain the observed decrease in KCASH2 levels through a decrease in Sp1 levels. Interestingly, we have observed a decrease in Sp1, and consequentially of KCASH2 protein, following DOXO exposition in HEK293T cells (Supplementary Figure 6).

MMA treatment reduces Sp1 and KCASH2 protein levels in WT MEF, as expected, but interestingly increases also p53 activity (Figure 6C). This observation is coherent with a previous report by Rao and colleagues in malignant pleural mesothelioma cells (Rao et al., 2016).

To better understand the interplay between Sp1 and p53, we analyzed the effect of MMA treatment in MEF p53^–/–^. As expected, drug treatment reduces Sp1 protein levels, but surprisingly, we observed a concomitant increase in KCASH2 protein (Figure 6D). Conversely, in the same cell line, the ectopic expression of Sp1 leads to a decrease in KCASH2 expression (Figure 6E).

To verify whether this seemingly paradoxical effect was restricted to MEF cells (although we observed that the p53- and Sp1-mediated regulatory mechanisms were apparently conserved from mouse to human), we analyzed the interplay of the two TFs in a human cell model carrying mutant p53.

To this purpose, we used human MB DAOY cells, which harbor mutant p53 (p53^*C*252F^), which is unable to bind the promoter of target genes (Waye et al., 2015). In DAOY cells, Sp1 overexpression leads to a reduction in reporter activity (Figure 6F) and KCASH2 protein levels (Figure 6G), an effect analogous to that observed in MEF p53^–/–^. Similarly, the reduction of Sp1 activity, following MMA treatment, results in an increase in mRNA (Figure 6H) and protein levels of KCASH2 (Figure 6I).

We further confirmed these observations in human cervical tumor HeLa cells, in which p53 expression is strongly repressed by overexpression of E6 protein from oncogenic HPV type 16 (Hoppe-Seyler and Butz, 1993). Also, in this case, the ectopic Sp1 expression leads to a decrement in KCASH2 protein levels (Supplementary Figure 7).

Our data seem to suggest that Sp1 mediates KCASH2 transcriptional repression in cells lacking p53 functionality, indicating that the availability of p53 determines if Sp1 acts as a transcription activator or repressor, as previously reported in different models and target genes (Innocente and Lee, 2005; Lin et al., 2010), although the mechanisms of action are not clear.

### DNA Methylation Downregulates KCASH2 Expression in Cells Lacking p53 Activity

It may be possible that, in the absence of p53, epigenetic modifiers are transcribed and recruited to make chromatin less accessible and Sp1 sites less exposed as well, preventing binding from TFs. Indeed, p53 not only enforces the genomic stability but also plays a role in regulating the epigenetic changes that can occur in cells (Levine and Berger, 2017).

Of note, the expression of the DNA 5′-cytosine-methyltransferases (DNMT1) has been shown to be modulated by both p53 and Sp1 in lung cancer cells (Lin et al., 2010). Indeed, while high levels of Sp1 induce DNMT1 expression, p53 complexing with Sp1 negatively regulates its expression (Lin et al., 2010). Interestingly, in p53-null cells, ectopic Sp1 induces higher DNMT1 expression, indicating that DNMT1 deregulation is associated with a gain of transcriptional activation of Sp1 and/or loss of p53 repression (Lin et al., 2010).

**FIGURE 7:**
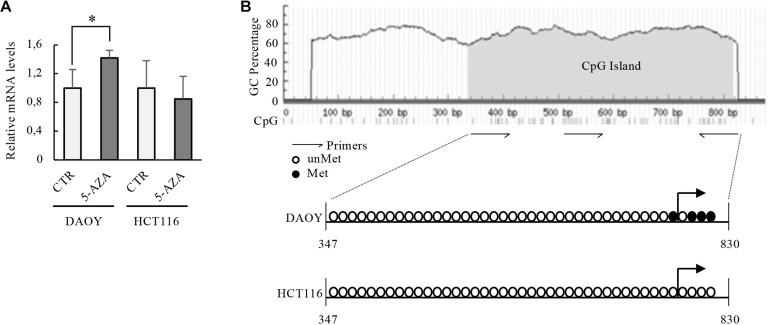
Transcriptional activity of KCASH2 promoter in p53 mutated and WT tumor cell lines. **(A)** p53 mutant DAOY and p53 WT HCT116 cells were treated with 5-AZA (5 μM). Then, KCASH2 mRNA levels were analyzed by RT-qPCR, normalized to Actin, and represented as fold induction on CTR. Bars represent the mean of three independent experiments ± SD.^∗^*p* < 0.05. **(B)** Methylation level of the CpG island on the KCASH2 proximal promoter. Genomic DNA was purified from DAOY and HCT116 cells and frequencies of CpG islands with and without methylation were measured by sequencing after bisulfate treatment. Methylation statuses of CpG dinucleotides are marked as non-methylated (open circle) and methylated (closed circle). Among marked regions, the methylation level was measured as a ratio of methylated CpG to total CpG. DAOY cells resulted methylated (10% CpGs), instead, HCT116 cells resulted totally unmethylated. The gray area on the schematic representation of the CpGs island indicate the CpG island, the dashes at the bottom of the sequence represent single CpG dinucleotides.

Given the previous considerations, we investigated if DNA methylation could be a regulatory mechanism on KCASH2 promoter activity. In fact, we identified a putative CpG island, which contains 49 CpG dinucleotides, overlapping most of the KCASH2 promoter (from −360 to 120 bp, see also Figure 7).

To evaluate whether methylation regulates the access of transcription factors on KCASH2 promoter, we first investigated the effects of demethylating agent 5′-Aza-2′-deoxycitidine (5-AZA) treatment on KCASH2 transcription in DAOY cells. Indeed, we previously described KCASH2 as a suppressor of Hh signaling, and its loss was able to reduce the Hh pathway activity and Hh-dependent DAOY cell proliferation (De Smaele et al., 2011; Spiombi et al., 2019).

The treatment of DAOY cells with 5-AZA, a drug that induces selective degradation of DNMT1 (Ghoshal et al., 2018), significantly increases KCASH2 mRNA levels (Figure 7A), suggesting that in these cells, there is an inhibition of KCASH2 expression driven by DNMT1. Of note, hypermethylated DAOY cells presented lower basal KCASH2 reporter luciferase activity compared with HEK293T (Supplementary Figure 8).

On the other hand, in colon cancer HCT116 cells (p53 WT), KCASH2 mRNA levels do not undergo significant changes after 5-AZA treatment (Figure 7A), suggesting that DNA methylation participates in KCASH2 transcription suppression when p53 is lost.

To support this observation, we therefore analyzed the methylation profile of KCASH2 promoter in DAOY and HCT116 cell lines. Sequencing of DNAs extracted after sodium bisulfite treatment show that KCASH2 promoter is differentially methylated in DAOY compared with HCT116 cells (Figure 7B). In fact, 10% of CpG dinucleotides of CpG island result in methylated p53-mutated DAOY cells compared with p53 WT HCT116 cells, in which ipomethylation was observed. Most interestingly, the only methylated CpGs of the proximal KCASH2 promoter are located close to the transcriptional start site (TSS). Taken together, these data demonstrate a direct role of methylation in KCASH2 transcriptional activity in p53-deficient cells.

Although further experiments need to be performed in order to fully demonstrate the mechanisms of action, we could hypothesize that in p53-mutated DAOY tumor cells, Sp1 may downregulate KCASH2 expression through DNMT1induction.

## Discussion

The KCASH2 promoter does not have a typical TATA-box but presents several GC-boxes, typical binding motifs for Sp1 (Yang et al., 2007). Sp1 has been shown to drive the expression of a wide plethora of human TATA-less genes either ubiquitously expressed or undergoing complex developmental and cell-specific regulation (Gross and Oelgeschläger, 2006).

Together with Sp1, computational analysis on the human KCASH2 promoter has identified several putative BS for other transcription factors, including SMAD, KIF, NeuroD, AP2alpha, and p53 (see Supplementary Figure 2). Of these, we investigated the role of p53 in the regulation of the KCASH2 promoter primarily because of its extraordinary relevance of p53 in tumorigenesis. The hypothesis that p53 may play a role in KCASH2 modulation would have been very interesting since KCASH2 is also a tumor suppressor in the Hedgehog-dependent tumor context. Indeed, p53 also plays a role in Hedgehog-dependent tumorigenesis: mouse models suggest that the loss of p53 may be associated to increased frequency of Hh-dependent tumorigenesis (Wetmore et al., 2001), and TP53 mutations are enriched among SHH medulloblastomas (Zhukova et al., 2013). Interestingly, a good number of these potential binding sites is conserved in highly homologous regions between human and mouse KCASH2 promoters, suggesting that this gene may be regulated in a conserved way in both organisms.

The work presented here is focused on the highly represented and conserved pattern of regulation mediated by Sp1 and the oncosuppressor p53.

Sp1 is a well-characterized transcriptional activator (Bouwman and Philipsen, 2002). It is essential for proper expression of a large variety of genes involved in development, cell growth regulation, and cancer (Black et al., 2001; Lomberk and Urrutia, 2005). Moreover, it is responsible for recruiting TATA-binding protein and fixing the TSS at TATA less promoters (Blake et al., 1990; Black et al., 2001) and as such plays a role on the TATA-less KCASH2 promoter.

Indeed, overexpression of Sp1 leads to an increase in KCASH2 transcription and protein levels. Conversely, the treatment with MMA, a selective well-known Sp1 inhibitor, reduced KCASH2 transcription and protein.

The single contribution of Sp1 BS to KCASH2 activity may depend also on the distance between them, which can determine the availability for activators or co-repressor to interact with Sp1, mediating KCASH2 regulation (Koutsodontis et al., 2001).

Among the eight putative Sp1 BS, Sp1B, Sp1C, and Sp1F seem to play a significant role: their mutation reduced by 50% the transcriptional activity of the KCASH2 promoter. Of note, mutation of the Sp1C site alone induced a dramatic reduction (> 90%) in transcriptional activity, although it was still able to weakly respond to Sp1 overexpression, probably because of the remaining active sites. On the other hand, mutation of Sp1D induced an increase in the transcriptional activity, which may indicate a somewhat repressive role for this site. We hypothesize that Sp1 binding on the C site may favor the activation of the transcriptional machinery, while binding on the D site may either induce recruitment of repressor protein or sterically interfere with the functionality of the transcriptional complex present on the C site. Moreover, Sp1 may interact with multiple factors simultaneously, particularly when present as a multimer, resulting in a large number of complexes and, consequentially, in a different activation or repression of target gene promoters. Another potential explanation is that Sp3, a Sp1-parolog, which has been described to act as a transcriptional repressor, may bind preferentially to BS-D. Indeed, the two TFs recognize similar, if not the same, DNA sequences (Völkel et al., 2015) and may compete for binding, thus Sp3 can repress KCASH2 gene expression by binding to the D site.

Another possibility was that the D site may be occupied by other factors such as p53 that, in our context, acts as negative regulator of KCASH2 transcription, but results from oligonucleotide pulldown assays suggest that this is not the case.

Finally, we could not exclude that the single-site mutation, which is able to disrupt Sp1 binding, could also interfere with DNA topology and chromatin accessibility to chromatin modifiers that play a role in promoter regulation and transcription initiation.

Of interest, combined mutation of the most active Sp1 BS demonstrates an increased inhibitory effect on transcription, while the mutation of all the BS (except for the H site) lost the responsivity to Sp1 overexpression, although its basal level was not completely turned off.

Sp1 overexpression has been linked to tumorigenesis and to altered expression of a number of essential oncogenes and tumor suppressors (Beishline and Azizkhan-Clifford, 2015). It is interesting to note that Sp1 expression was found high also in Hh-dependent MB tumor cells (Eslin et al., 2013), while KCASH2 expression was reduced in MB cells (De Smaele et al., 2011). For this reason, we could have expected an inverse correlation between the two genes’ expressions. This apparent paradox is shared with the other KCASH family member KCASH1, whose activity increases following Sp1 overexpression (Mancarelli et al., 2010). We hypothesize therefore that Sp1 may contribute to the assembly of the transcription complex and sustain KCASHs basal level of activity, which could be further enhanced or downregulated by other TFs or by epigenetic modifications.

On the other side, while we have demonstrated that KCASH2 has at least one functional site for p53 binding, we would have expected that p53 overexpression would have a positive effect on KCASH2 transcription, in agreement with the oncosuppressor role of both proteins.

We observed, on the contrary, that p53 downregulates KCASH2 expression.

Indeed, p53 may act also as a repressor; although the mechanism of action has not been unanimously agreed, there are several genes that are repressed following overexpression or activation of p53 (Ho and Benchimol, 2003; Lin et al., 2010; Fischer et al., 2015, 2016). Several models of p53 action have been proposed, involving interference with activators, interference with basal machinery, and action on modifiers (e.g., recruitment of HDACs and reduced accessibility to the promoter) (Ho and Benchimol, 2003). Indeed, p53 may specifically interact with and recruit corepressors such as the mSin3A–HDAC complex or SMRT. It has been recently suggested that p53 repression may be through a mechanism involving the DREAM complex or RB (Engeland, 2018; Uxa et al., 2019). Nevertheless, in our hands, mutation of the putative p53 BS on the KCASH2 promoter abolishes such repression, implying that binding of p53 on the p53 BS is required. These data are also confirmed by the ChIP assay, which indicates the recruitment of p53 on the promoter.

According to the presence of a Sp1 BS (Sp1B) close to the p53 BS, we may hypothesize that the mechanism of action of p53 implies the interaction between these two transcription factors, as has been previously suggested (Lin et al., 2010). Indeed, we have shown that DOXO treatment can activate p53, while downregulating Sp1 and KCASH2 protein levels.

The complex crosstalk between p53 and Sp1 warrants further study. Intriguingly, p53 and Sp1 share similar consensus sequences at GC-boxes along the human genome, suggesting that they might interplay in transcription regulation and may even compete in binding to specific promoters or function in opposite directions (Oppenheim and Lahav, 2017). It has also been suggested that some of the inhibitory activity of p53 is exerted through downregulation of Sp1 (Jun et al., 2017). Another possibility that we cannot exclude is that, in our context, p53 does not directly exert a true repressory effect, but may have a weaker transcriptional activity compared with Sp1. In this scenario, p53 binding on the promoter may simply interfere with Sp1 binding to some of the Sp1 BS and substitute a strong transcriptional activity with a weaker one, resulting in an overall reduction in KCASH2 transcription.

At the same time, we have demonstrated that treatment with MMA may increase p53 levels; this is in agreement with Phillips et al., who suggested that MMA may stabilize p53 at post-transcriptional level, by negative modulation of MDM2 mRNA nuclear export and its translation (Phillips et al., 2006).

On the other hand, Sp1 and p53 may form a complex, together with chromatin modifiers, inducing repression of the DNMT1 gene (Hsu et al., 2014). Moreover, p53 can sequester Sp1, preventing its interaction with target sequences (Bocangel et al., 2009). Interestingly, whenever it happens, a shift in the balance between the two factors, either by an increase in Sp1 or a decrease in p53, will enhance DNMT1 gene expression (Hsu et al., 2014). Based on our data, we may hypothesize that recruitment of the p53 protein on KCASH2 promoter, close to an Sp1 site, may allow the formation of a complex acting as a transcriptional modulator.

The most intriguing results presented here indicate that in the absence of an active p53 protein, either by knock out (in p53^–/–^ MEF), by mutation of the gene (in DAOY cells), or by post-translational interference on p53 (HeLa cells), Sp1 appears to play a suppressive role on the KCASH2 promoter. Coherently, reduction in Sp1 activity by MMA induced a KCASH2 increase.

Tumor suppressor genes (e.g., p16 and VHL) are frequently silenced by DNA methylation of their promoters (Jones and Baylin, 2002). Interestingly, Sp1 and p53 modulate DNMT1 and, in a p53 null context, Sp1 expression leads to an increase in DNMT1 (Lin et al., 2010) and by consequence promoter methylation of target genes.

**FIGURE 8:**
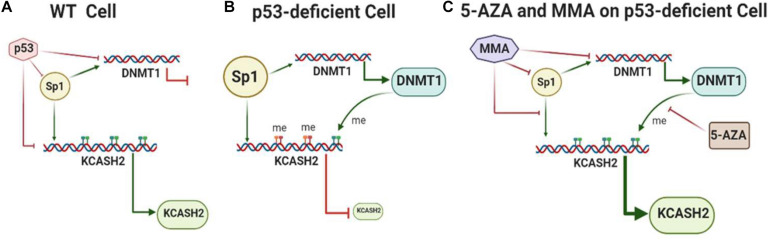
Model of KCASH2 regulation in WT and p53-deficient cells. **(A)** In WT cells, transcriptional activity of the KCASH2 promoter is balanced by the effect of basal transcription sustained by Sp1 and suppression operated by p53. In this context, p53 also inhibits Sp1-dependent DNMT1 transcription, leaving the KCASH2 promoter accessible to transcription factors. **(B)** In p53-deficient cell, Sp1 is upregulated and leads to active DNMT1 transcription. The consequent methylation on CpG islands of the KCASH2 promoter inhibits Sp1 binding and its capability to activate KCASH2 transcription, resulting in low levels of KCASH2. **(C)** MMA and 5-AZA treatment in p53-deficient cells enhance KCASH2 transcription. MMA reduces Sp1 levels, and its binding and activation of the DNMT1 promoter. 5-AZA treatment leads to demethylation of the CpG island rendering KCASH2 promoter available for Sp1 binding and Sp1-mediated basal activation.

We may hypothesize a model (Figure 8) (which will need to be confirmed by further evidences) according to which, in WT cells, basal KCASH2 expression is balanced by the effects of transcription induced by Sp1 and suppression operated by p53. On the other hand, in cellular and tumor models in which p53 is lost, mutated, or inactivated, the expression of Sp1 is enhanced, leading to an increase in DNMT1 protein. DNMT1 is therefore responsible for promoter methylation, turning off KCASH2 expression, since Sp1 is not able to bind the methylated GC on the promoter and sustain basal levels of KCASH2. Coherently with this model, DAOY cells exhibit higher levels of KCASH2 promoter methylation and increase KCASH2 transcription following treatment with the demethylating agent 5-AZA (Patties et al., 2013).

Recently, new molecules able to modulate the Hh signaling have been designed and developed for MB treatment (Quaglio et al., 2020). However, drug resistance and tumor relapse remain the greatest challenge, making it necessary to find further new therapeutic strategies (Cowman et al., 2019).

Given the role of KCASH2, which acts downstream of the Hh pathway, to the level of Gli1 transcription factor, the capability to modulate its expression may add a new therapeutic tool. Interestingly, some authors have already suggested the use of Sp1 inhibitors, alone or in combination with a standard chemotherapeutic drug for MB treatment (Eslin et al., 2013; Patil et al., 2019).

According to our model, it would be a promising therapeutic approach to enhance KCASH2 expression in MBs, by the use of Sp1 inhibitors in combination with demethylating agents and other Hh inhibitors acting on different targets. This would be particularly effective in the treatment of Hh-dependent MB characterized by p53 deficiency.

## Data Availability Statement

The original contributions presented in the study are included in the article/Supplementary Material, further inquiries can be directed to the corresponding author/s.

## Author Contributions

AA and ADF designed and performed the majority of the experiments, analyzed the results, and wrote the manuscript. EDS and MM originated, conceived, and supervised the project, and wrote the manuscript. CADT, SF, MP, LLS, FB, and LB performed the experiments and contributed to the data analysis. EF and GC discussed the results and provided critical reagents and comments. All authors critically reviewed and approved the article before submission.

## Conflict of Interest

The authors declare that the research was conducted in the absence of any commercial or financial relationships that could be construed as a potential conflict of interest.

## Footnotes

1 www.epidesigner.com

2 http://genome.ucsc.edu/

3 http://genexplain.com/transfac

## Abbreviations

KCASH2: KCTD-containing Cullin3 Adaptor Suppressor of Hedgehog 2
Hh: Hedgehog
MB: Medulloblastoma
MEF: Mouse Embryonal Fibroblasts
MMA: Mithramycin
DOXO: Doxorubicin hydrochloride
5-AZA: 5-Aza2′deoxycytidine
TSS: Transcription Start Site
BS: Binding Site
Sp1: Specific protein 1
TF: transcription factor
WT: wild type
KO: knock-out
DNMT1: DNA 5′- Cytosine-Methyltransferase
SD: standard deviation
ChIP: Chromatine Immunoprecipitation.
