# Supplementary material for: Specific Protein 1 and p53 Interplay Modulates the Expression of the KCTD-Containing Cullin3 Adaptor Suppressor of Hedgehog 2
Source: Front Cell Dev Biol. 2021 Apr 8;9:638508. doi: 10.3389/fcell.2021.638508 (PMC8060498; doi:10.3389/fcell.2021.638508)
Supplement: Supplementary file 1 [file Data_Sheet_1.ZIP › Angrisani et al. Figure S1.pdf]

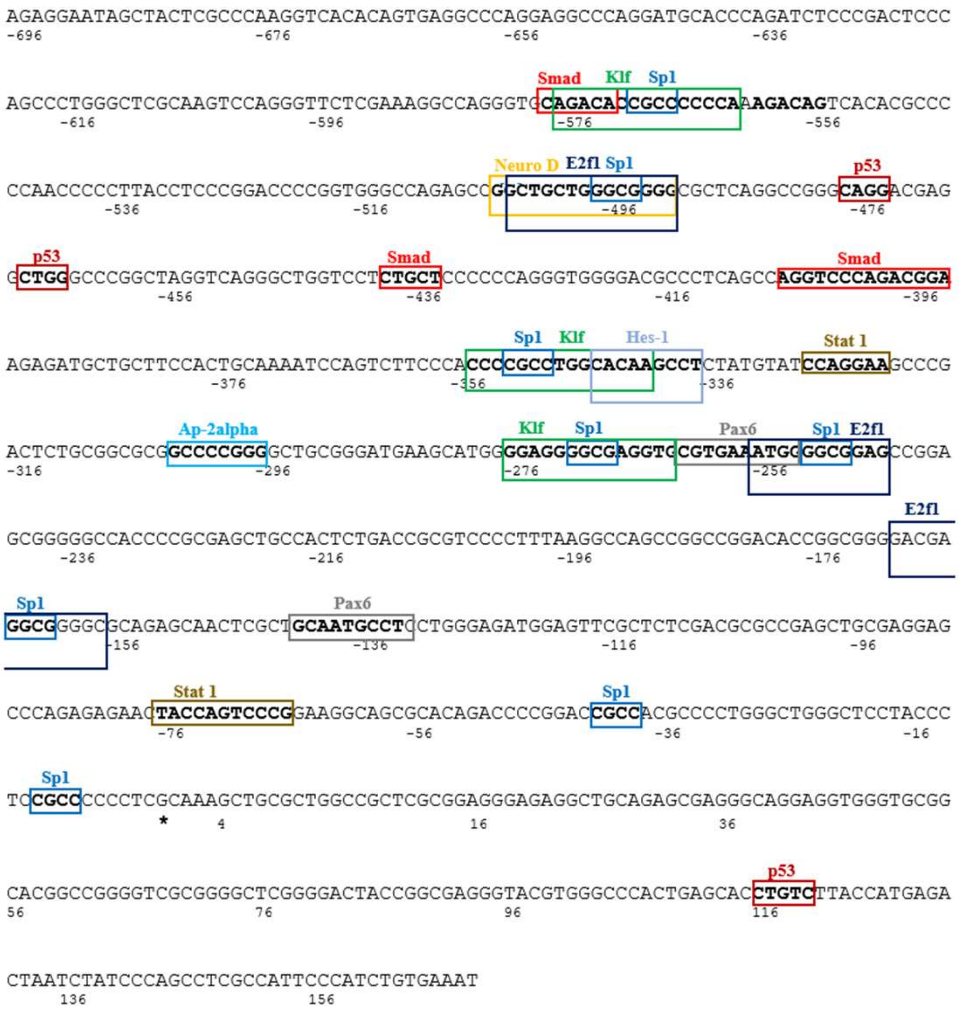

Angrisani et al. Figure S1
