# Supplementary material for: Specific Protein 1 and p53 Interplay Modulates the Expression of the KCTD-Containing Cullin3 Adaptor Suppressor of Hedgehog 2
Source: Front Cell Dev Biol. 2021 Apr 8;9:638508. doi: 10.3389/fcell.2021.638508 (PMC8060498; doi:10.3389/fcell.2021.638508)
Supplement: Supplementary file 1 [file Data_Sheet_1.ZIP › Angrisani et al. Figure S3.pdf]

```
human       AGAGGAATAGCTACTCGCCCAAGGTCACACAGTGAGGCCCAGGAGGCCCAGGATGCACCC    60
mouse       ------------------------------------------------------------    0

human       AGATCTCCCGACTCCCAGCCCTGGGCTCGCAAGTCCAGGGTTCTCGAAAGGCCAGGGTGC    120
mouse       ------------------------------------------------------------    0

                  Sp1-A
human       AGACACCGCCCCCAAAGACAGTCACACGCCCCCAACCCCCTTACCTCCCGGACCCCGGT    180
mouse       ------------------------------------AACTCCTAGACTTCAGAGGGCCTT    24
                                                  * * **     * **   *  *
                              Sp1-B               p53-A
human       GGGCCAGAGCCGGCTGCTGGGCGGGGCGCTCAGGCCGGGCAGGACGAGGCTGGGCCGGC    240
mouse       ACCTTAGAACCCACTCCTGGGCGGGGCGCATGGGCCGGACTAGACTAGGCTGGGCCAGTC    84
                 *** **  ** *************   ****** *  *** ********** * *

human       TAGGTCAGGGCTGGTCCTCTGCTCCCCCCAGGGTGGGGACGCCCTCAGCCAGGTCCCAGA    300
mouse       GAGGTCAGGGCGGGTCCTCCGCTCCCCTACCGAGG----------------GGTCCCAGA    128
             ********** ******* *******    *  *                *********
                                                      Sp1-C
human       CGGAAGAGATGCTGCTTCCACTGCAAAA---TCCAGTCTTCCCACCCCGCCTGGCACAAG    357
mouse       CCGCAGGGATGCTGCTGCTACTTTGTGATGTCCAACTCCACCCACCCCGCTCCGCACAAC    188
            * * ** ********* * ***     *    * * **  **********   ******

human       CCTCTATGTATCCAGGAAGCCCGACTCTGCGGCGCGGCCCCGGGGCTGCGGGATGAAGCA    417
mouse       CCTAGATGGACCTGGGCAGTGCAACTGC-----GCGGTCTCAGAGCTGCGGGACGGAACT    243
            ***  *** * *  ** **  * ***       **** * * * ********* * * *
                    Sp1-D               Sp1-E
human       TGGGGAGGGGCGAGG-TGCGTGAAATGGGGCGGAGCCGGAGCGGGGGCCACCCCGCGAGC    476
mouse       GGGAGAGGGGTGAGGTGCGGGAAACGGGGCGGAGCCACAGCGGGGGTTACCCTGCGAGC    303
             ** ****** ****    * **** ***********  ********  **** ******
                                                                 Sp1-F
human       TGCCACTCTGACCGCGTCCCCTTTAAGGCCAGCCGGCCGGACACCGGCGGGGACGAGGCG    536
mouse       TCCCAGTCGGGCCGCGTCCCCTTTAAGGCCAGCTGGTCCCAGTGCCGGCGGGGCGGGCCA    363
            * *** ** * ********************** ** *  *   * *  *** ** * *

human       GGGCGCAGAGCAACTCGCTGCAATGCCTCCTGGGAGATGGAGTTCGCTCTCGACGCGCCG    596
mouse       G-TAGGGGGCTGGCTCTCCGCGTAGCCTCCTGGGAGGTGGAGTCCGGGCCCGGCACGCGG    422
            *   *  *     *** * **   ************ ****** **  * ** * *** *

human       AGCTGCGAGGAGCCCAGAGAGAACTACCAGTCCCGGAAGGCAGCGCACAGACCCCGGACC    656
mouse       CACAGGAAGGAACGCAGAGGGGACTACAAGTCCCGGCGGCCAGCGCGCGGCCCGGGACGA    482
              * *  **** * ***** * ***** ********  * ****** * * **  *
Sp1-G                             Sp1-H
human       GCCACGCCCCTGGGCTGGGCTCCTACCCTCCGCCCCCCTCGCAAAGCTGCGCTGGCCGCT    716
mouse       CCCGCC--CCTG------CCCCAGCCCGGCCCACCCCCTAGCGAAGCTGCGC-------T    527
             ** *   ****       * *   **  **  ****** **  *********       *

human       CGCGGAGGGAGAGGCTGCAGAGCGAGGGCAGGAGGTGGGTGCGGCACGGCCGGGGTCGCG    776
mouse       GGCTGGAGGGGAGGCTGCCGAGCGAGGGCAGGAGGTGGGTGCGGTGCGCAGGGGTTTCGG    587
             ** * **  ******** *************************  **   *** *   *
                                                        p53-B
human       GGGCTCGGGGACTACCGGCGAGGGTACGTGGGCCCACTGAGCACCTGTCTTACCATGAGA    836
mouse       GGAGGGCTCTGGACCCAGCGGACGCATA-----------------CATGTC----------    621
            **            ** ***   * *                 * ****

human       CTAATCTATCCCAGCCTCGCCATTCCCATCTGTGAAAT    874
mouse       --------------------------------------    621
```

Angrisani et al. Figure S3
