# Supplementary material for: Specific Protein 1 and p53 Interplay Modulates the Expression of the KCTD-Containing Cullin3 Adaptor Suppressor of Hedgehog 2
Source: Front Cell Dev Biol. 2021 Apr 8;9:638508. doi: 10.3389/fcell.2021.638508 (PMC8060498; doi:10.3389/fcell.2021.638508)
Supplement: Supplementary file 1 [file Data_Sheet_1.ZIP › table 1.pdf]

NAME	OLIGO SEQUENCE (5'-3')
KCASH2 promoter FW	AGCTCTCGAGAGAGGATAGCTACTCGCCC
KCASH2 promoter RV	CGCGAAGCTTATTTCACAGATGGGAATGGCG
Sp1 BS_Mut A FW	GGTGCAGACACCGCCCCCCAAAGACACAC
Sp1 BS_Mut B FW	GAGCCGGCTGCTGGGCGGGGCGCTCAGGCC
Sp1 BS_Mut C FW	CTTCCCACCCCGCCTGGCACAAGCCTC
Sp1 BS_Mut D FW	GAAGCATGGGGAGGGGCGAGGTGCGTGAAATG
Sp1 BS_Mut E FW	GTGCGTGAAATGGGGCGGAGCCGGAG
Sp1 BS_Mut F FW	GGCGGGGACGAGGCGGGGCGCAGAG
Sp1 BS_Mut G FW	CGGACCGCCACGCCCCTGGGCTGGG
Sp1 BS_Mut H FW	CTCCTACCCTCACCCCCCCTCGCAAAG
Sp1 BS_Mut H RV	CTTTGCGAGGGGGGGTGAGGGTAGGAG
P53 BS_Mut A FW	CGCTCAGGCCGGGAACGAGGCTGG
P53 BS_Mut A RV	CCAGCCTCGTTCTTCCCGGCCTGAGCG
Promo Met 10 FW	TTTGGTATAAGTTTTTATGTATTTAGGAA
Promo Met 11 FW	GTAATGTTTTTTGGGAGATGGAGTT
Promo Met 10/11 RV	AATAAAACAAATACTCAATAAACCCAC
promok21sp1-C Wt FW probe	Biotin-CTTCCCACCCCGCCTGGCACAAGC
promok21sp1-C Wt RV probe	Biotin-GCTTGTGCCAGGCGGGGTGGGAAG
promok21sp1-D Wt FW probe	Biotin-CATGGGGAGGGGCGAGGTGCGTGA

promok21sp1-D Wt RV probe	Biotin-TCACGCACCTCGCCCCTCCCCATG
promoPrimerChip FW	CTTCCACTGCAAAATCCAGTCTTC
promoPrimerChip RV	CAGAGTCGGGCTTCCTGGATAC

Table 1.
