# Supplementary material for: Specific Protein 1 and p53 Interplay Modulates the Expression of the KCTD-Containing Cullin3 Adaptor Suppressor of Hedgehog 2
Source: Front Cell Dev Biol. 2021 Apr 8;9:638508. doi: 10.3389/fcell.2021.638508 (PMC8060498; doi:10.3389/fcell.2021.638508)
Supplement: Supplementary file 1 [file Data_Sheet_1.ZIP › supp fig captions.pdf]

SUPPLEMENTARY FIGURES LEGENDS

Figure S1: Representation of putative TFs binding sites on KCASH2 proximal promoter.
The recognized sites for TFs are highlighted in the rectangles. TSS is specified with the asterisk. The sequence is numbered relative to TSS.

Figure S2: Basal activity of the proximal KCASH2 promoter.
HEK293T cells were transfected with KCASH2 promoter-Luc reporter and empty-Luc reporter. The luciferase activity was normalized to Renilla Luciferase activity. Data are presented as mean of three independent experiments ± standard deviation (SD), ***p<0,001.

Figure S3: Sp1 and p53 BS are conserved across human and mouse promoter KCASH2 sequences.
Alignment of KCASH2 proximal promoter across human and mouse sequences.
* represents a matched nt.

Figure S4: P53 reduces H4 acetylation (acH4) in KCASH2 promoter.
Cross-linked chromatin was extracted from HEK293T cells treated for 5h with Doxorubicin 5 µM and immunoprecipitated with a relevant control IgG or specific anti-AcH4 antibody. Immunoprecipitated chromatin samples were analyzed by RT-qPCR using KCASH2 promoter selective primers. Relative enrichment was calculated by Delta CT analysis and it is expressed as fold induction of treated versus not treated.

Figure S5: p53 does not recognize Sp1 BS C-D DNA regions.
(A) Western Blot of the oligo pulldown assay. The pulldown was performed with Dynabeads magnetic beads added with 400µg of Nuclear extract and 4µg of doublestrand-biotinylated-oligonucleotide.
(B) HEK293T were transfected with WT or MutC or MutD luciferase reporter together with an empty vector or a p53 expressing vector. Lysates were analyzed by luciferase assays. ***p<0,001; **p<0,01. Transfection efficiency in luciferase experiments was normalized by co-transfection of a pRL-TK-Renilla reporter. Data are representative of three independent experiments performed in triplicate and presented as mean ± SD.

Figure S6: DOXO treatment induced Sp1 degradation in HEK293T cells.
HEK293T cells were treated with DOXO (5 μM) for 24h. Protein lysates were analyzed through Western Blot. The p53 activation was verified with an antibody against phosphorylated Ser 15. Anti-vinculin was used as loading control.

Figure S7: Sp1 overexpression leads to decreased KCASH2 protein levels in p53 low expressing HeLa tumor cells.
HeLa cells were transfected with empty vector or Sp1 expressing plasmid, then analyzed by Western Blot. Tubulin was used as loading control.

Figure S8: KCASH2 basal transcriptional activity is lower in DAOY cells than in HEK293T.
DAOY and HEK293T cells were transfected with the KCASH2 promoter luciferase reporter. Luciferase activity is normalized to the control. Transfection efficiency in luciferase experiments was normalized by co-transfection of a pRL-TK-Renilla reporter. Data represented mean of three independent experiments ± standard deviation (SD). ***p<0,001; **p<0,01.

Supplementary Table S1: List of oligos used for cloning, mutagenesis, Chip, methylation and oligo pulldown.
