# Supplementary figures and images for: Specific Protein 1 and p53 Interplay Modulates the Expression of the KCTD-Containing Cullin3 Adaptor Suppressor of Hedgehog 2
Source: Front Cell Dev Biol. 2021 Apr 8;9:638508. doi: 10.3389/fcell.2021.638508 (PMC8060498; doi:10.3389/fcell.2021.638508)

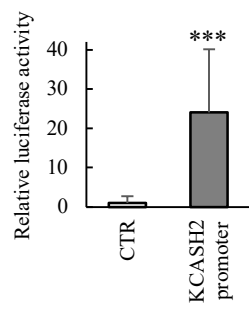

Supplement: Supplementary file 1 [file Data_Sheet_1.ZIP › Angrisani et al. Figure S2.pdf]

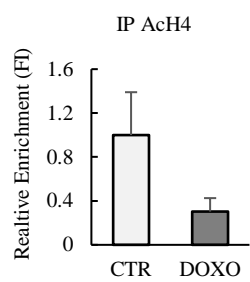

Supplement: Supplementary file 1 [file Data_Sheet_1.ZIP › Angrisani et al. Figure S4.pdf]

**A**

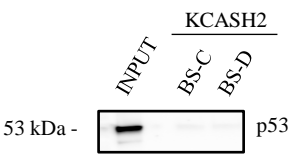

**B**

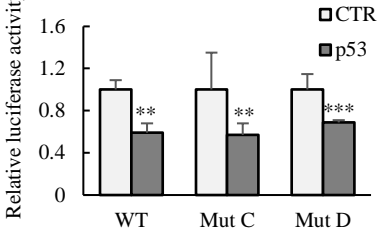

Supplement: Supplementary file 1 [file Data_Sheet_1.ZIP › Angrisani et al. Figure S5.pdf]

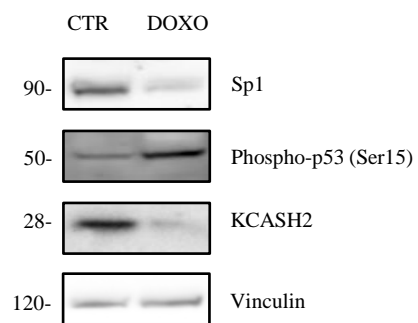

Supplement: Supplementary file 1 [file Data_Sheet_1.ZIP › Angrisani et al. Figure S6.pdf]

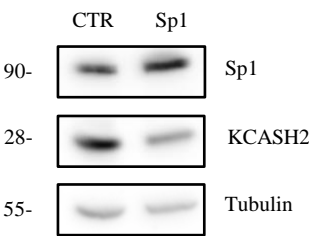

Supplement: Supplementary file 1 [file Data_Sheet_1.ZIP › Angrisani et al. Figure S7.pdf]

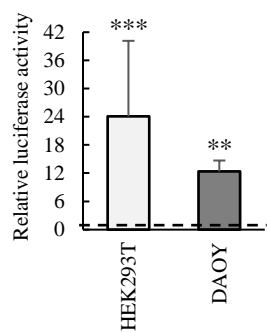

Supplement: Supplementary file 1 [file Data_Sheet_1.ZIP › Angrisani et al. Figure S8.pdf]
